# Irritable Bowel Syndrome and Risk of Parkinson’s Disease in Finland: A Nationwide Registry-Based Cohort Study

**DOI:** 10.3233/JPD-202330

**Published:** 2021-04-13

**Authors:** Tuomas H. Mertsalmi, Anna But, Eero Pekkonen, Filip Scheperjans

**Affiliations:** a Department of Neurology, Helsinki University Hospital and Department of Clinical Neurosciences (Neurology), University of Helsinki, Helsinki, Finland; b Biostatistics consulting, Department of Public Health, University of Helsinki and Helsinki University Hospital, Helsinki, Finland

**Keywords:** Parkinson’s disease, irritable bowel syndrome, gastrointestinal tract, epidemiology

## Abstract

**Background:**

The gastrointestinal tract is considered as a potential origin of Parkinson’s disease (PD) pathology. Besides constipation, appendectomy and inflammatory bowel disease have also been associated with a higher PD-risk, but findings have been inconsistent. To date, there is only one previous study suggesting that irritable bowel syndrome (IBS) is associated with an increased risk of PD.

**Objective:**

To evaluate whether IBS is associated with a higher risk of PD.

**Methods:**

In this retrospective registry-based cohort study, we identified 28,150 patients that were diagnosed with IBS (IBS+) during the years 1998–2014, using data from the Finnish Care Register for Health Care. In addition, 98,789 IBS-free reference subjects (IBS-) of same age and gender and living in the same municipality were included. The study subjects were followed until the end of the year 2014 to analyze the incidence of PD. The association between IBS and PD was assessed by a Cox proportional hazards model.

**Results:**

Diagnosis of IBS was associated with a higher hazard of PD with an adjusted hazard ratio (aHR) of 1.70 (95% CI 1.27–2.26). However, the ratio of hazard rates for PD between IBS+ and IBS- subjects was not constant over time. The Cox model with time-varying coefficient for IBS status showed that the hazard of PD was significantly higher in IBS patients only during the first two years of follow-up (aHR 2.96, 95% CI 1.78–4.92).

**Conclusion:**

Our findings indicate that the association between IBS and PD is likely explained by reverse causation and detection bias. It remains open whether IBS is an actual risk factor or a prodromal symptom of PD.

## INTRODUCTION

Patients with Parkinson’s disease (PD) may suffer from multiple non-motor symptoms, including gastrointestinal dysfunction, hyposmia, and REM sleep behavior disorder. With a reported prevalence of up to 80%, constipation is considered the most common non-motor symptom in PD, emerging up to 20 years before the onset of motor symptoms [1]. It is also recognized as a significant risk factor for PD [2, 3]. The gastrointestinal tract shows widespread changes in the enteric nervous system already in the earliest stages of PD [4–6]. The early involvement of the gastrointestinal tract has led to a hypothesis that the pathological process of PD might initiate in the gut [7]. Indeed, several factors associated with a higher risk of developing PD, such as exposure to metals and pesticides, or antibiotics could induce alpha-synuclein misfolding by toxic effects on the gastrointestinal tissues and/or microbiome [8, 9]. Alpha-synuclein, the pathological hallmark for PD, has prion-like properties and it can spread along the vagus nerve towards the central nervous system [10]. In support of this theory, a truncal vagotomy seems to protect against PD [11].

In addition to constipation, PD patients suffer from a variety of other gastrointestinal symptoms, including dysphagia, and impaired gastric emptying [12]. Recently, we showed that in PD patients, when specifically assessed using the Rome-criteria[13], symptoms of irritable bowel syndrome (IBS) were more prevalent than functional constipation and were associated with changes in faecal microbiota [14]. IBS is a disorder of gut-brain interaction characterized by abdominal pain or discomfort, and alteration of bowel habits [15]. The prevalence of IBS in Finland is 5.1% according to the Rome II criteria [16]. IBS has been connected with a higher risk of PD in the Taiwanese population, but these findings have not yet been reproduced elsewhere [17]. Besides IBS, also appendectomy [18] and inflammatory bowel disease (IBD) [19–22] have been associated with a higher PD-risk, but these findings have not been consistent. Regarding IBD, it has been suggested that the positive association could be explained by surveillance bias, also known as detection bias [23]. We designed a nationwide registry-based cohort study to evaluate whether IBS diagnosis is associated with a higher risk of PD in Finland.

## MATERIALS AND METHODS

**Fig. 1:**
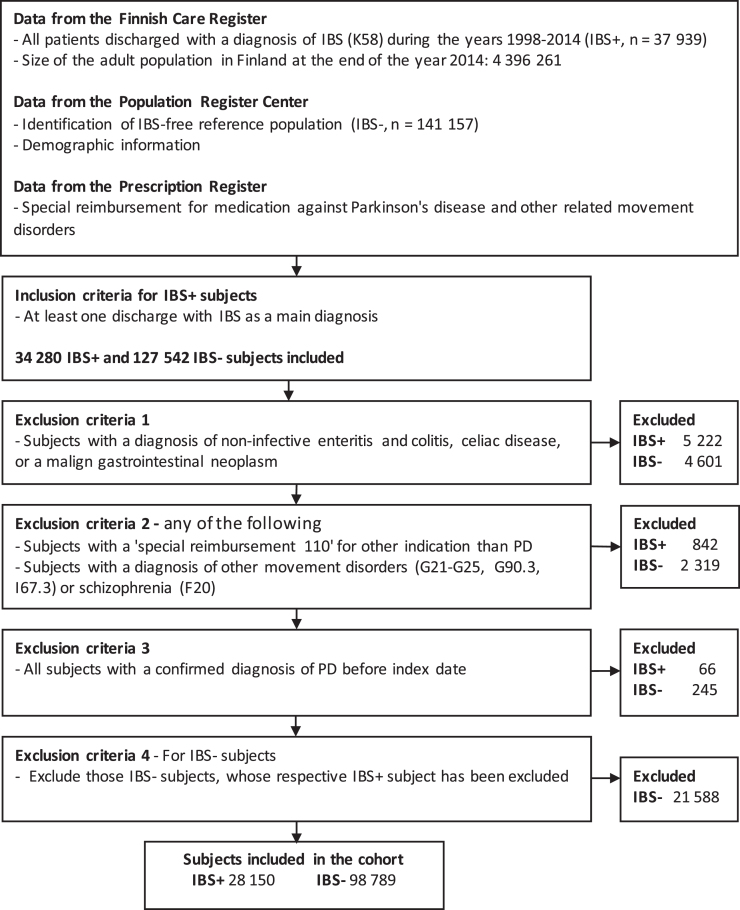
Study flowchart representing data collection and exclusion criteria of the study

In this registry-based observational follow-up st-udy, we used several registries to form an IBS cohort (IBS+), to select a reference IBS-free (IBS-) population, and to identify those who were diagnosed with PD during follow-up. The Finnish Care Register for Health Care (HILMO), established in 1969, contains data on all discharges from inpatient care, day surgeries, and, since 1998, specialized outpatient care [24]. It is linkable by personal identification codes and includes diagnostic codes (according to the International classification of diseases, 10th version, ICD-10). The diagnostic codes used for this study are explained in Table 1. All adult patients (aged 20 years or older) that were discharged from inpatient care or specialized outpatient care with IBS as a main diagnosis (ICD-10 code K58, including subcategories) during the years 1998–2014 were identified and included in the initial cohort (Fig. 1). At the end of the year 2014, the size of the adult population in Finland was 4,396,261 [25]. The index date was the date of the first discharge with IBS-diagnosis. For each IBS patient (IBS+), up to four IBS- reference subjects of the same age (±1 year), sex and place of residence were acquired from the Population Register Center. The information about possible emigration or death during the follow-up period were also acquired from the Population Register Center. All discharge data of IBS+ and IBS- subjects were acquired from the HILMO-register.

**Table 1:** ICD-10 codes used for identification of irritable bowel syndrome patients, patients with Parkinson’s disease or exclusive diagnoses, and potential confounders

**Irritable bowel syndrome**	K58
**Parkinson’s disease**	G20
**Exclusive diagnoses**
Crohn’s disease	K50
Ulcerative colitis	K51
Other noninfective gastroenteritis and colitis	K52
Celiac disease	K90.0
Colorectal neoplasms	C18, C19, C20, C21, C78.5, D01
Schizophrenia	F20
Secondary parkinsonism	G21
Parkinsonism in diseases classified elsewhere	G22
Other degenerative diseases of basal ganglia	G23
Dystonia	G24
Other extrapyramidal and movement disorders	G25
Multiple system atrophy	G90.3
Progressive vascular leukoencephalopathy	I67.3
**Potential confounders**
Depression	F32, F33, F34.1
Anxiety disorders	F40, F41, F43, F06.4
Chronic obstructive pulmonary disease	J44

^a^If only the three-character category is listed, also its subcategories were included. Some variation may occur in the contents of the ICD-10-codes regionally.

In Finland, the PD-diagnosis is usually set by neurologists in the public secondary or tertiary healthcare units. However, sometimes a neurologist working in the private sector that is not included in the HILMO-register, can set the PD-diagnosis first. If so, several years may pass before the patients visits pu-blic health care due to PD for the first time. Therefore, we used data from two different registers to identify patients with PD. Using data from the HILMO-reg-ister, we identified all patients that were discharged from inpatient care or specialized outpatient care with a diagnostic code of PD (G20) during the years 1998–2014. We also obtained data from the Finnish Nat-ional Prescription register, established in 1987, on whether subjects had been entitled for the special rei-mbursement of drug expenses for treatment of PD and comparable movement disorders (special reimbursement code 110, applicable for patients that have a diagnosis associated with following ICD-10 codes G20, G23, G24.1, G24.8, G90.3) between the years 1987-2014. Special reimbursement data between the years 1987–1997 was used only to exclude subjects who were probably diagnosed with PD before the establishment of the HILMO register. To be entitled to this special reimbursement 110 (SR-110), the diagnosis and need for treatment had to be certified by a neurologist or a doctor working in a neurology unit.

We considered the PD-diagnosis to be reliable if the subject had at least three discharges with a PD-diagnosis at a specialized neurology unit [26]. For those patients entitled to SR-110, two discharges from a specialized neurology unit with PD were con-sidered sufficient. The date of PD-diagnosis was de-fined as the first date when the patient was discharged with PD-diagnosis, or the date SR-110 was granted, whichever occurred earlier. The study population was retrospectively followed up starting from the index date until the occurrence of PD, emigration, death or the end of the follow-up period (Dec 31, 2014), whichever occurred first. Emigration, death and the end of the follow-up period without PD diagnosis were considered as censoring.

To reduce the possibility of a misdiagnosis of IBS and to exclude subjects with other major gastrointestinal disorders, all subjects with a discharge including a diagnosis of IBD or other non-infective enteritis or colitis, celiac disease, or colorectal neoplasms between the years 1998–2014 were excluded (Fig. 1). Also, all patients that had been entitled to SR-110 for an indication other than PD and those with a diagnosis of other movement disorders or schizophrenia before or after the index date were excluded from the study. Finally, we excluded all subjects with confirmed PD before the index date.

We identified potential confounders based on dire-cted acyclic graphs (Supplementary Figure 1) [27]. The history of depression, anxiety disorders, and chronic obstructive pulmonary disease (COPD) be-fore the index date were assessed by using the discharge data. Depression and anxiety disorders are more common in patients with IBS [28] and are also associated with a higher risk of PD [29]. Since data on smoking was not available, COPD was used as proxy for smoking, which is associated with a reduced risk of PD [30].

### Statistical analyses

The statistical analyses were performed using IBM SPSS Statistics version 25.0.0.1 (IBM Corp.) and R statistical software (version 3.6.2). For baseline characteristics, we used Fisher’s two-sided exact test for differences regarding dichotomous categorical variables. For normally distributed variables, an unpaired *t*-test was used to analyze the group differences of baseline characteristics; otherwise, the Mann-Whitney U test was used. *P*-values below 0.05 were considered significant.

In the primary analysis, the hazard ratios (HR) and 95% confidence intervals (CI) were assessed for the association between IBS and PD using univariate and multivariable Cox proportional hazard models with follow-up time as the underlying time scale. Age, gender, and history of COPD, depression, and anxiety disorders were included in the multivariable model. We accounted for the clustered structure of the data by assessing robust standard errors for HR. To check the validity of the proportional hazards assumption, we performed a visual assessment of the complementary log-log plots and assessed the possible interaction between IBS status and follow-up time in the Cox model. No significant interaction was found between IBS status and follow-up time (*p* = 0.344) suggesting that the coefficient for IBS status as a function of time follows no simple functional form.

We evaluated how the ratio of hazard rates for PD between IBS+ and IBS- subjects behaved over time by estimating the hazard rate of each group by two-year intervals using the life table method. The estimated hazard rate of each of the studied groups was visualized with a piecewise constant step function, suggesting that the hazard rate ratio of two groups was not constant over time. Based on this, we analyzed the hazard of PD in three separate time periods (0–2, >2–5, and over 5 years) using the Cox model with time-varying coefficients.

To evaluate how sensitive our findings were to changes in the definition of exposure (IBS) and event (PD), we performed two additional analyses using the Cox model with a time-varying coefficient for IBS status: 1) with stricter inclusion criteria, demanding at least two discharges with IBS-diagnosis and 2) with a more liberal definition of PD (entitlement to SR-110 due to PD). Furthermore, to assess the consistency of the results across men and women and by age groups, we assessed sex-specific HRs and performed analysis by restricting to the older age groups at baseline. Finally, to assess the potential role of death as a competing risk, we studied mortality during the follow-up by analyzing the hazard of death in three time periods (0–2,>2–5, and over 5 years) similarly to the PD-hazard analysis.

### Standard protocol approvals

The study design was approved by the Data Protection Ombudsman from the Ministry of Social Affairs and Health and by all the register authorities who have provided data for the research. Ethical approval is not required for registry studies in Finland. Identifiers enabling a direct identification of individuals were replaced with research identification codes to maintain patient confidentiality. The study was conducted in accordance with the Strengthening the Reporting of Observational Studies in Epidemiology recommendations [31].

## RESULTS

After applying exclusion criteria (Fig. 1), there were 28150 IBS+ and 98789 IBS- subjects, women being over-represented in the latter group (Table 2). In contrast, depression, anxiety disorders, and COPD were more common in IBS+ than IBS- subjects. During a median follow-up of 6.3 years (IQR 2.9–9.9), a total of 186058 and 651041 person-years accumulated in IBS+ and IBS- subjects, respectively. 70 IBS+ and 148 IBS- subjects were diagnosed with PD during the follow-up, yielding the incidence of 3.76 and 2.27 per 10000 person-years, respectively. During the follow-up, 109 (0.4%) of IBS+ subjects and 483 (0.5%) of IBS- subjects were censored due to emigration, whereas 1936 (6.9%) and 7396 (7.5%) were censored due to death, respectively. In 94.8% of PD-cases, the special reimbursement for PD-medication was granted within two years of the first discharge with PD-diagnosis.

**Table 2:** Baseline characteristics and incidence of Parkinson’s disease

	IBS +	IBS –
Number of subjects	28 150	98 789
Females, No. (%)	20114 (71.5)	71615 (72.5)	**0.001**
Age, median [IQR]	50.69 [36.67–63.27]	50.70 [36.57–63.34]	0.928
**Potential confounders**
Depression, No. (%)	2771 (9.8)	4423 (4.5)	<0.001
Anxiety disorders, No. (%)	1882 (6.7)	2911 (2.9)	<0.001
COPD, No (%)	412 (1.5)	956 (1.0)	<0.001
**Follow-up and incidence of PD**
Follow-up years, median [IQR]	6.33 [2.87–9.98]	6.29 [2.90–9.91]	0.612
Hospitalizations, median [IQR]	1 [0–3]	1 [0–2]	**<0.001**
Outpatient visits, median [IQR]	10 [4–25]	4 [1–12]	**<0.001**
PD, No. (%)	70 (0.2)	148 (0.1)	**0.001**
Incidence per 10000 person years	3.76	2.27
Age at PD-diagnosis, median [IQR]	69.85 [62.79–75.52]	72.06 [65.93–78.14]	0.147
Time from index-date to PD-diagnosis, median [IQR]	3.03 [1.12–7.85]	4.60 [2.38–7.25]	0.169

COPD, chronic obstructive pulmonary disease; IBS, irritable dowel disorder; IQR, interquartile range; PD, Parkinson’s disease; SD, standard deviation.

**Fig. 2:**
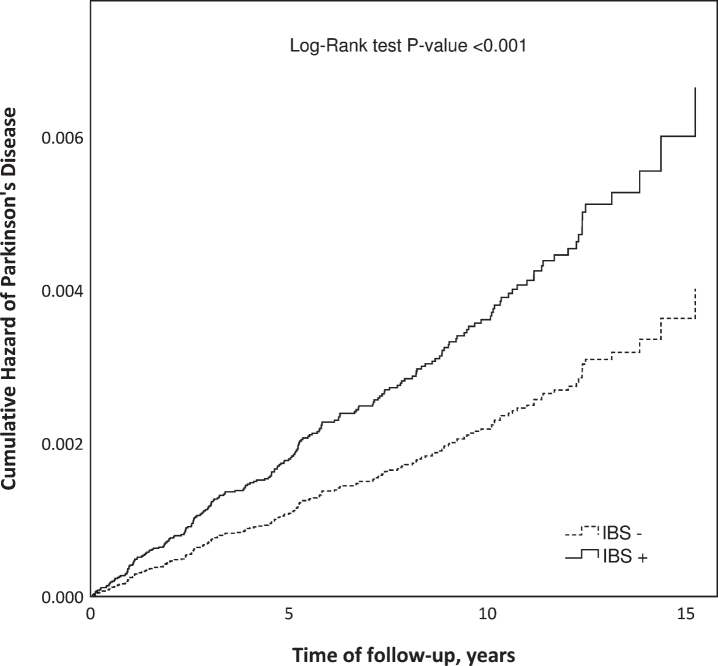
Cumulative hazard of Parkinson’s disease in patients diagnosed with irritable bowel syndrome (IBS+) compared to an IBS-free reference population (IBS–) matched by gender, age, and place of living.

In the univariate Cox model, the HR for PD was 1.65 (95% CI 1.24–2.20) for IBS+ subjects compared with IBS- subjects (Fig. 2). When potential confounders (depression, anxiety disorders, COPD, age and gender) were included in the model, the adjusted HR (aHR) for PD was 1.70 (95% CI 1.27–2.26).

**Fig. 3:**
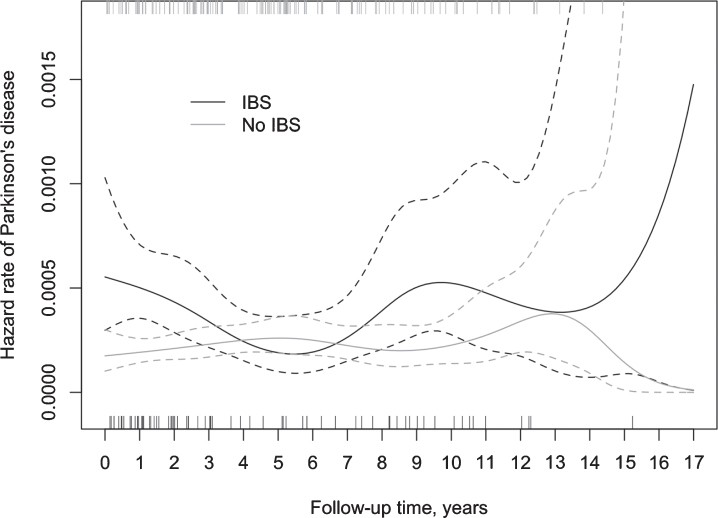
Incidence of PD (spikes) in IBS+ and IBS–subjects during the follow-up and the corresponding hazard rate, including 95% confidence intervals.

The results of the life table analysis suggested a considerable variation in the ratio of hazard rates for PD between IBS+ and IBS- subjects over time, mostly due to a substantial variation in the hazard rate for PD among IBS patients (Fig. 3). In the IBS- group, the hazard rate for PD remained relatively stable during the whole follow-up period. The Cox model with a time-varying coefficient for IBS status (Table 3) showed the highest hazard ratio during the first two years of follow-up (aHR 2.96, 95% CI 1.78–4.92). Between two and five years of follow-up, the relative difference diminished, whereas beyond the follow-up of five years the hazard ratio demonstrated an increase, though not statistically significant.

**Table 3:** Incidence of Parkinson’s disease, hazard ratios, associated 95% confidence intervals, and sensitivity analyses

	N	Events (%)	0–2 years		2–5 years		over 5 years	
			Crude HR [95% CI]	Adjusted HR^a^ [95% CI]	Crude HR [95% CI]	Adjusted HR^a^ [95% CI]	Crude HR [95% CI]	Adjusted HR^a^ [95% CI]
All	126939	218 (0.2)	2.88 [1.73–4.78]	2.96 [1.78–4.92]	1.05 [0.59–1.88]	1.08 [0.61–1.93]	1.50 [0.96–2.33]	1.53 [0.98–2.38]
At least 2 discharges with IBS	43211	73 (0.2)	6.44 [2.38–17.40]	6.70 [2.48–18.16]	1.31 [0.51–3.36]	1.36 [0.53–3.48]	1.44 [0.69–3.02]	1.48 [0.71–3.09]
More liberal definition for PD^b^	126939	373 (0.3)	2.27 [1.45–3.57]	2.30 [1.46–3.61]	1.06 [0.68–1.65]	1.08 [0.69–1.67]	1.50 [1.09–2.06]	1.51 [01">1.10–2.07]
Mortality	126939	9332 (7.4)	1.00 [0.91–1.10]	NA	0.90 [0.83–0.99]	NA	0.88 [0.81–0.95]	NA

CI, confidence interval; IBS, irritable bowel syndrome; HR, hazard ratio; PD, Parkinson’s disease; SR-110, special reimbursement for PD medication. ^a^Adjusted for age, gender, depression, anxiety disorders, and chronic obstructive pulmonary disease. ^b^Only a special reimbursement for PD-medication (SR-110) was required, regardless of the discharge data.

### Sensitivity analyses

With stricter inclusion criteria for IBS definition, we identified only 9,572 IBS subjects and only 73 PD cases were observed in the whole cohort during the follow-up. Although the hazard of PD was substantially higher during the first two years of follow-up, statistical power was limited in the analyses regarding the later follow-up years due to the smaller sample sizes and numbers of events (Table 3). With a more liberal definition of PD, analysis using the Cox model with a time-varying coefficient for IBS status showed similar results to the primary analyses although the aHR reached statistical significance after five years of follow-up. Stratification by gender showed that the risk of PD for those with IBS was higher in women (aHR 2.22, 95% CI 1.56–3.16), but not in men (aHR 1.01, 95% CI 0.60–1.70). Subgroup analyzes of different age groups lacked statistical power. However, restricting analysis to subjects aged at least 50 years at the index date provided results similar to those of the main analysis (data not shown). The hazard rate of death was lower in IBS+ subjects compared with IBS- subjects, the differences being apparent already between two and five years of follow-up (HR 0.90, 95% CI 0.83–0.99) and remaining at least at the same level after five years of follow-up (HR 0.88, 95% CI 0.81–0.95).

## DISCUSSION

In this observational register-based follow-up stu-dy on the large cohort of IBS patients and an IBS-free reference population, we found an association bet-ween IBS and an increased hazard of PD only during the first two years of follow-up. During this period almost a 3-fold hazard rate was seen among IBS+ subjects compared with IBS- subjects. At least partly, these results may suggest that IBS patients are in relatively close surveillance before IBS-diagnosis and during the first years after it, and therefore their pot-ential premotor or early motor symptoms are more likely to become detected by a clinician. This is referred as detection bias in epidemiological res-earch. Since non-motor symptoms of PD can precede the onset of motor symptoms by several years [32], it is also possible that IBS is a manifestation of pro-dromal PD. Patients with early PD might first seek medical care for their gastrointestinal symptoms and might therefore be first diagnosed with IBS. PD dia-gnosis was established later when their motor symptoms became more prominent. Therefore, at least to some extent, the association between IBS and being diagnosed with PD during the first two years of fol-low-up could also be explained by reverse causation. Reverse causation has been suggested to at least partly explain the associations found between PD and certain protective or risk factors, such as smoking, coffee drinking, exercise, and plasma urate levels [33]. IBS patients had a decreased hazard of death during the follow-up, indicating that to some extent, the difference in the hazard of PD between IBS+ and IBS- subjects could also be explained by competing risks bias when considering long-term association. The effect of death as a competing risk on the observed risk for PD in epidemiologic studies has earlier been demonstrated by Driver and colleagues [34].

In a previous study on the Taiwanese population, IBS was associated with a higher hazard for PD with an adjusted HR of 1.48 (95% CI 1.27–1.72) [17]. Consistently with our data, the HR for PD was the highest during the first two years of follow-up (aHR 1.77, 95% CI 1.33–2.36), suggesting detection bias and possible reverse causation. In the study on Tai-wanese population, the hazard of PD remained significantly higher for IBS patients also in those with over two years of follow-up (aHR 1.38, 95% CI 1.16–1.66). However, detection bias may extend past ten years of follow-up [35].

Our findings indicate a lack of association between IBS and PD after the first two years of follow-up. In part, this could also be explained by the statistical power, which tends to vary over time and impedes the reliable evaluation of long-term associations. In fact, in the present study the statistical power of the primary analysis was insufficient to detect with 80% power relative differences smaller than 2.02 after five years of follow up. In the sensitivity analysis with more liberal PD-definition the smallest HR detectable with 80% power was 1.66. Of note, the hazard ratio for PD in patients with constipation was 3.03 in a recent Danish population-based study, and the pooled OR 2.27 in a meta-analysis performed by Adams-Carr et al. [2, 3]. Therefore, we were able to detect similar relative differences in the hazard rate as have been observed with constipation. Stratification by gender showed that the hazard of PD was higher in female IBS+ subjects as compared to IBS- females (aHR 2.22, 95% CI 1.56–3.16) but not in IBS+ males as compared to IBS- males (aHR 1.01, 95% CI 0.60–1.70). However, the analysis of males may have lacked statistical power due to a smaller sample size. Of note, no gender-specific differences in the PD hazard were observed in the Taiwanese population [17].

Many factors linked to IBS, such as low-grade inf-lammation, increased gut permeability, and disturba-nces of the neuroendocrine system [36], could make the gastrointestinal tract more vulnerable to external toxins and pathogens, thereby making it a more probable initiation site for alpha-synuclein aggregation. Microbiome alterations have been described in IBS and PD [37, 38] and these changes might affect the mucosal binding of pathogens and mucosal barrier function [36, 39]. Interestingly, microbiome alterat-ions are particularly pronounced in PD patients that suffer from IBS-symptoms [14, 40] implicating microbiota as a link between these two disorders. It is possible that there is a true association between IBS and PD. However, more studies are needed with longer follow-up and careful consideration of possible reverse causation, detection bias, competing risks and controlling for confounding to further investigate the possible association.

The strengths of our study include a large nationwide cohort including individuals of wide age range. The cohort in this study—formed by using nationwide registers—was representative of the IBS pop-ulation. Therefore, the results of this study provide the risk of PD in Finnish IBS population, given that bias is not introduced [41]. We have evaluated the possible effect of different sources of bias by adjusting for several confounders and performing sensitivity analyses. Observational studies are prone to bias due to confounding, but adjustment for factors other than confounders may introduce bias as well. Therefore, it is important to carefully consider which factors are the most likely to be a source of confounding. Selecting potential confounders is extremely tricky in a disorder like PD, where the pathogenesis is com-plex and only partially known. After a careful consideration using directed acyclic graphs [27], we selected several potential confounders (Supplementary Material) and controlled for their effect through adjustment. In addition, we exhaustively assessed the proportional hazards assumption and detected a substantial variation in both the rate and ratio of hazards over time. The Cox proportional hazard model relies on the assumption of the hazard ratio being constant over time, which should be carefully assessed [42]. Moreover, to gain better understanding about the phenomenon under study, not only variation in the hazard ratio but also in the hazard rates of interest should be inspected.

As a typical of observational studies [41], alongside the advantages there are also limitations and disadvantages to consider regarding the present study. The data of the HILMO-register (previously named the Finnish Hospital Discharge Register) has not been collected primarily for the purposes of specific research questions, but when its validity has been studied, the positive predictive value for common diagnoses has been between 75–99% [24]. However, the validity of IBS- and PD-diagnoses has not been specifically investigated, and therefore the misclassification of both exposure and outcome of interest cannot be ruled out entirely in the lack of clinical confirmation. In sensitivity analyses, we investigated the robustness of our findings with respect to different criteria for the definition of IBS- and PD-cases and found the results to be robust, although the statistical power was limited regarding the analyses with stricter inclusion criteria for IBS patients.

Because the diagnosis of IBS can take a long time, we selected reference subjects from those without IBS diagnosis at the index date but also during the years 1998–2014. This might have introduced selection bias. Indeed, those without IBS diagnosis during the follow-up period are likely to differ from IBS patients with respect to the factors that are related with chances of or can preclude from being diagnosed with IBS. These factors include use of or access to health care services and death. In this study, IBS+ subjects had more outpatient visits and experienced death at a lower rate compared with IBS- subjects, suggesting a limited comparability of the studied groups. In fact, confounding by some of the unmeasured characteristics underlying the abovementioned differences cannot be ruled out.

There are also other limitations to consider. We had limited information on whether the IBS-diagnoses were made according to the gold standard, Rome criteria [13]. IBS diagnosis involves ruling out other gastrointestinal diseases and there are certain ‘red flag symptoms’ that need to be considered before ruling out other possible diagnoses. Our exclusion criteria included celiac disease, IBD, and cancer, but some of the ‘red flag symptoms’, such as anemia, night sweats, or hypersedimentation were not assessed. This could lead to the misclassification of gastrointestinal symptoms. It is possible that in the IBS-group there are subjects that suffered from rather unspecific abdominal discomfort that could not be attributed to other causes and thus received the IBS diagnosis without actually being assessed specifically using the Rome criteria [13]. We demanded a discharge with IBS as a main diagnosis, to reduce the possibility that the diagnosis was based solely on a subjects’ own notion. In Finland, IBS is typically diagnosed by a general practitioner at the health care center. Since the data from outpatient visits at the health centres was not available for this study, it is likely that some of the IBS- subjects in this study actually have IBS, but the information about the diagnosis is missing from the HILMO-register. Would there be IBS patients among the reference population, it cannot be exclu-ded that this might have disturbed the observed association between IBS and PD. Based on the estimated prevalence of IBS in Finland (5.1%) and the size of the adult population (ca. 4.4 million) it can be calculated that roughly 200,000–250,000 adults in Finland have IBS, of which 11–14% were included in our IBS+ cohort. Given these numbers, approximately 5,000 subjects in the IBS- group actually had IBS, which might have affected the observed association (crude HR 1.65) between IBS and PD in either direction, but the magnitude of this effect was rather small. We estimated that, depending on whether these subjects represent IBS with milder symptoms and have the same incidence of PD as other reference subjects, or have the same incidence of PD as those in the IBS+ group, 7–12 of the PD cases observed in the IBS- group would actually belong to the IBS+ and the crude HR for PD would be 1.55–1.72 for IBS patients as compared to the IBS-free population. Furthermore, the reference population was selected from those who did not have any discharges with IBS diagnosis during the whole study period. This might have introduced selection bias as those who were free of IBS diagnosis at the index date were precluded of becoming diagnosed with IBS during the follow-up. By applying this criterion, we aimed at avoiding misclassification bias with respect to IBS. Thus, it can be seen as a trade-off solution between potential misclassification bias and potential selection bias.

To consider the uncertainty of PD diagnosis at a single visit and clear recording errors that could introduce misclassification bias, we demanded multiple discharges with PD-diagnosis and combined the data with information on special reimbursements for PD medication. We also used strict exclusion criteria for secondary parkinsonism. We evaluated the onset of PD by considering both the date of the first discharge with PD-diagnosis and the date when special reimbursement for PD-medication was granted. In 94.8% of PD-cases in this study, the SR-110 was granted within two years of the first discharge with a PD-diagnosis. However, we did not have information on the actual onset of non-motor and motor symptoms and there is a possibility of delay in diagnosis. This might also partly explain some of the association observed between IBS and PD during the first two years of follow-up. At the end of follow-up, still the majority (75.4%) of subjects were under 70 years old, whereas the median age at the time of PD-diagnosis was 71.2 years in our cohort. It is possible that a subset of the subjects developed PD after the end of follow-up. In the present study the median follow-up time of 6.3 years (IQR 2.9–9.9) is in line with the duration of follow-up times in other studies investigating the association between IBS (6.2 years) or IBD (3.5–9.3 years) and PD [17, 19–21]. However, longer follow-up is needed to comprehensively cover the age range of the highest PD risk.

Although age and gender, which are strong confounders, were initially controlled by study design, females were over-represented in the IBS- reference group after the application of the exclusion criteria. Even when an equal distribution is achieved by matching, it does not completely solve the problem of confounding. It has been shown that bias can retain when ignoring the matching variables in cohort studies [43]. Therefore, we included age and sex in the multivariable analysis. In addition, depression, anxiety disorders and COPD were adjusted for in the multivariable analysis. Anxiety disorders and depression have been associated with a higher risk of PD although it can be argued that these are rather prodromal signs than true risk factors for PD. However, these were selected as potential confounders due to their association also with IBS. COPD was used as proxy for smoking. However, there might be some residual confounding due to unmeasured unknown factors that remained uncontrolled. For instance, the actual smoking status was not adjusted for, and we cannot exclude that this has affected our results. To evaluate the potential effect of residual confounding, we also calculated the E-value, which represents the required strength of association needed (to both the exposure and outcome) for an unmeasured confounder to nullify the observed association between IBS and PD in our data [44]. The estimated E-value of 2.79 suggests that the observed association between IBS and hazard of PD in the primary analysis were unlikely to be explained by a single unmeasured confounder.

Considering that subjects with IBS had on average more discharges from outpatient clinics and hospitals than IBS- subjects, and that according to validation studies the recording of subsidiary diagnoses has been poor, it is possible that the history of anxiety disorders, depression, and COPD have been registered more comprehensively for IBS patients. Some factors that affect the risk of PD, such as coffee consumption, BMI, dietary habits, physical activity, and exposure to pesticides, could not be assessed due to the study setting. Moreover, in the observational studies with long follow-up and confounders measured at the baseline only, estimates can be affected by residual confounding due to changes in confounders and their distributions over time. In fact, imbalance in confounders, when changing over time, cannot be controlled adequately through adjustment for baseline differences.

In conclusion, our findings suggest that previous evidence of an epidemiologic link between IBS and PD could be explained by reverse causation and detection bias during the initial years of follow-up, and/or death as a competing event when considering long-term association. The prodromal gastrointestinal symptoms of PD can be interpreted as symptoms of IBS. However, physiological changes caused by IBS could predispose the GI tract to PD pathology, and it is possible that there is a true association between IBS and PD, which could be detected with larger cohorts and longer follow-up. Carefully designed and statistically well-powered studies are needed to further investigate the possible short- and long-term associations between gastrointestinal disorders and PD. In future research, particular attention and effort should be given to the consideration and control of potential sources of bias both at design and analytical stages.

## CONFLICTS OF INTEREST

T.H.M., A.B, and F.S. reports no conflict of interest relative to the research covered in this manuscript. E.P. is a Member of the MDS Non-Motor Parkinson’s Disease Study Group.

## Supplementary Material

Supplementary Material
