# Supplementary material for: Irritable Bowel Syndrome and Risk of Parkinson’s Disease in Finland: A Nationwide Registry-Based Cohort Study
Source: J Parkinsons Dis. 2021 Apr 13;11(2):641–51. doi: 10.3233/JPD-202330 (PMC8150653; doi:10.3233/JPD-202330)

Supplementary Material

Irritable Bowel Syndrome and Risk of Parkinson’s Disease in Finland: A Nationwide Registry-Based Cohort Study

Supplementary Figure 1. Identification of potential confounding factors on the basis of directed acyclic graphs, assuming that the listed confounders’ effects via the gut-brain axis are minor compared to other mechanisms.

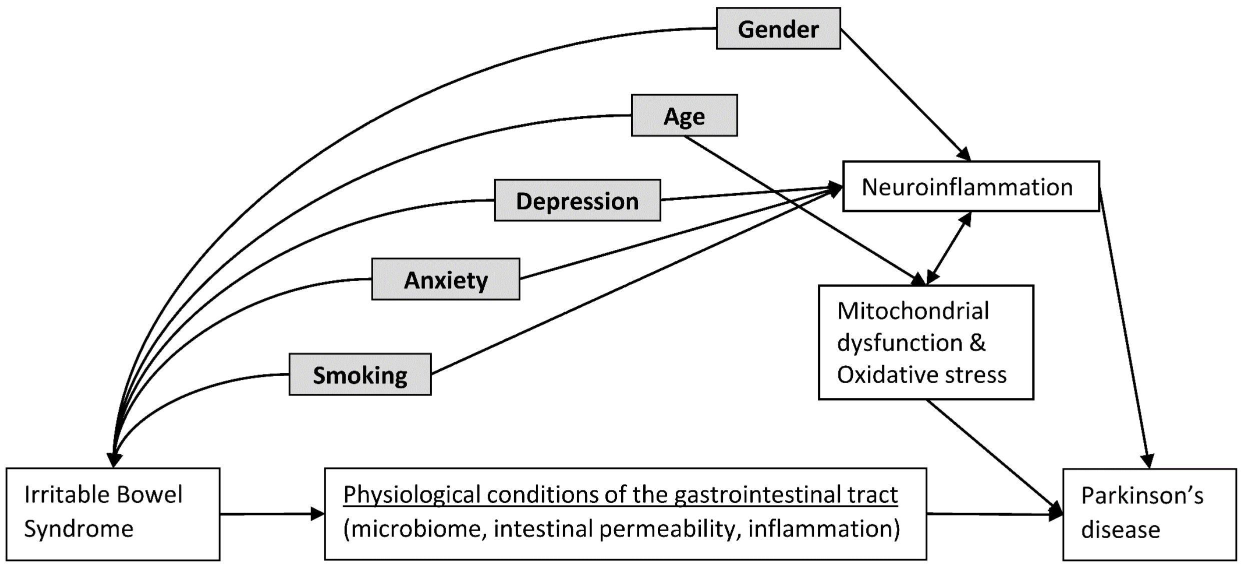

Supplement: Supplementary Material [file jpd-11-jpd202330-s001.pdf]
